# Endodontic Treatment and Esthetic Management of a Geminated Central Incisor Bearing a Talon Cusp

**DOI:** 10.1155/2014/123681

**Published:** 2014-03-05

**Authors:** Elif Tarım Ertaş, Meral Yırcalı Atıcı, Hakan Arslan, Bilal Yaşa, Hüseyin Ertaş

**Affiliations:** ^1^Department of Oral and Maxillofacial Radiology, Faculty of Dentistry, İzmir Katip Çelebi University, İzmir, Turkey; ^2^Department of Endodontics, Faculty of Dentistry, İzmir Katip Çelebi University, İzmir, Turkey; ^3^Department of Restorative Dentistry, Faculty of Dentistry, İzmir Katip Çelebi University, İzmir, Turkey

## Abstract

Gemination with talon cusps is an uncommon morphologic dental anomaly, characterized by the formation of clinically wide tooth that can cause significant aesthetic and clinical problems including esthetic impairment, pain, caries susceptibility, and tooth crowding. These morphological dental anomalies have specific treatment needs due to the abnormal morphology and need virtuous radiologic diagnosis. Multidisciplinary approach can supply success of the treatment plan that can provide esthetic and occlusal requirements. In this case report, the multidisciplinary approach for the treatment of geminated tooth with talon cusp is presented with the clinical and radiographic findings.

## 1. Introduction

Gemination is a rare morphological dental anomaly that develops when the single tooth bud attempts to divide to form two teeth. The anomalous tooth usually has totally or partially separated two crowns, with a single and large and maybe partially divided pulp chamber. In rare cases, division through the crown and root can be seen. Primary dentition is more frequently affected than the permanent dentition, usually in the incisor region [1]. The prevalence of gemination is variable and it generally ranges from 0.1 to 1% [2].

The etiology of gemination is unclear but there are several hypotheses like heredity, local metabolic interferences during morphodifferentiation of the tooth germ, environmental factors such as thalidomide embryopathy, fetal alcohol exposure, or hypervitaminosis A of the pregnant mother, and trauma [3].

Talon cusp is also a rare morphological dental anomaly of hyperplasia of the maxillary or mandibular incisor's cingulum, which is characterized by the presence of an accessory cusp-like structure. Talon cusp is usually seen in the cingulum area or cementoenamel junction of the mandibular or maxillary incisors both primary and permanent dentition and contains enamel, dentin, and also pulp tissue [4]. There is no predilection of sex and can be seen unilateral or bilateral. Its prevalence range is found to be 0.04–10% in various studies [5].

Talon cusp generally may occur isolated, but it can be very rarely associated with gemination. In the literature only six cases of geminated teeth with talon cusp have been reported [6]. The aim of this case report is to present the multidisciplinary approach for the treatment of geminated tooth with talon cusp with the clinical and radiographic findings.

## 2. Case Report

**Figure 1:**
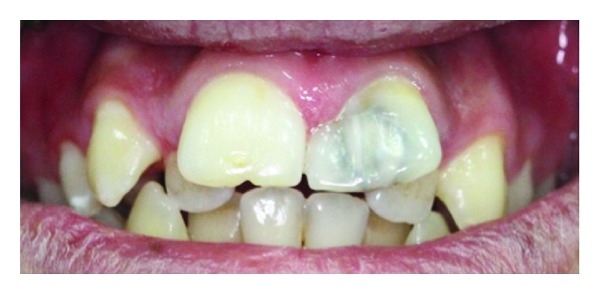
The intraoral photograph of bilateral wide central incisors.

**Figure 2:**
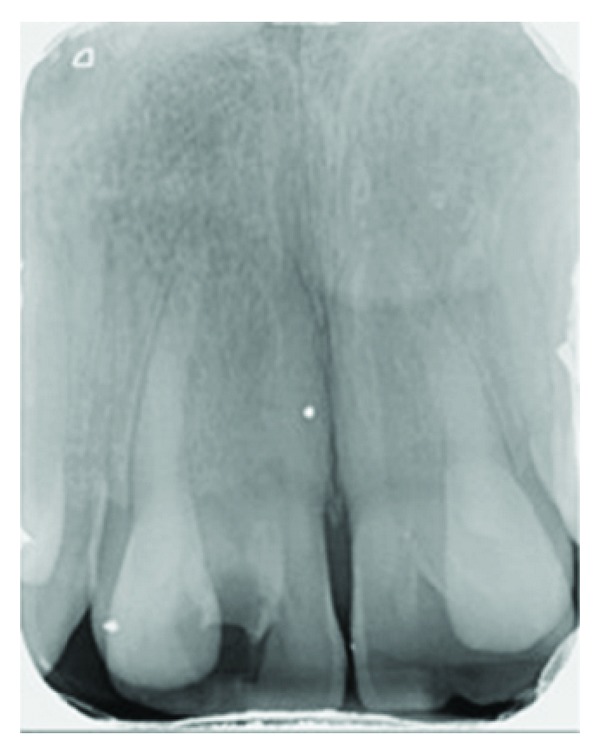
Periapical radiograph of the geminated incisors before treatment.

**Figure 3:**
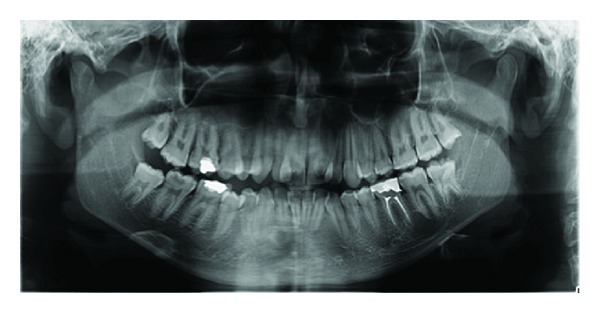
Panoramic radiographic image, which presents permanent dentition with complete teeth number.

**Figure 4:**
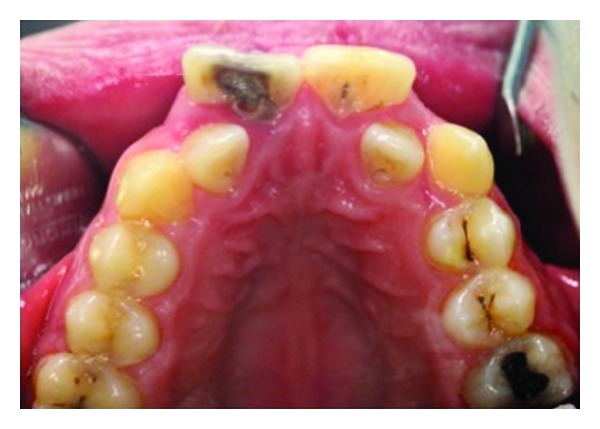
Talon cusp and deep carious lesion with pulp involvement on the palatal surface of the left central incisor.

**Figure 5:**
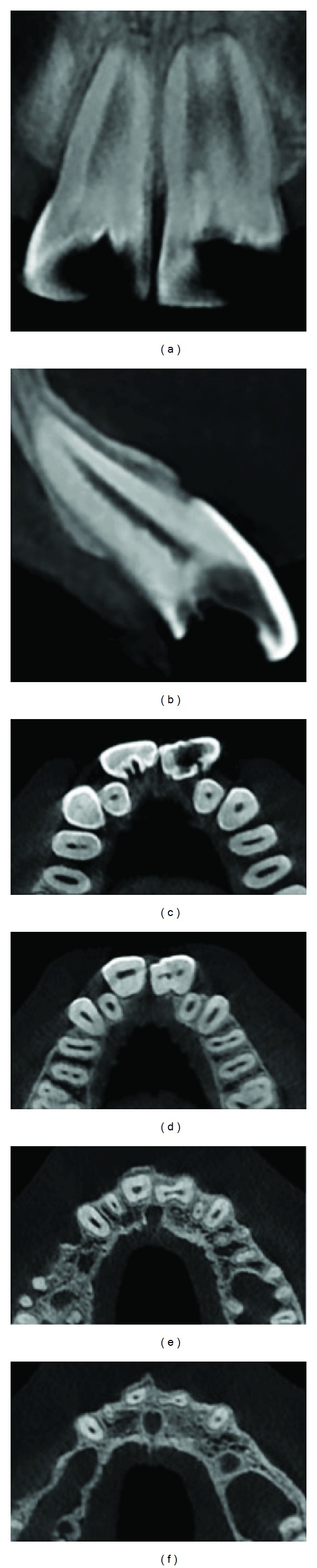
Different cone beam computed tomography (CBCT) sections of geminated incisors.

A 17-year old boy referred to our clinic with the complaints of pain and aesthetic problems. His medical history was noncontributory. On clinical and radiographic examination bilateral wide central incisors, crowding, and caries were determined (Figures 1 and 2). There was no missing tooth (Figure 3). With consideration of number of teeth, anomaly of central incisors was attributed to gemination. The permanent maxillary left central incisor had a large crown with talon cusp and deep carious lesion with pulp involvement on the palatal surface (Figure 4). He was consulted with endodontists and considered to perform cone beam computed tomography scan (CBCT) (New Tom 5G, Verona, Italy) to gain further insight into the root formation and canals. After obtaining informed consent form, CBCT was performed. Right maxillary incisor had a large pulp chamber and a large root canal while the left central incisor had a pulp chamber, which was dividing into mesial and distal two root canals (Figure 5).

**Figure 6:**
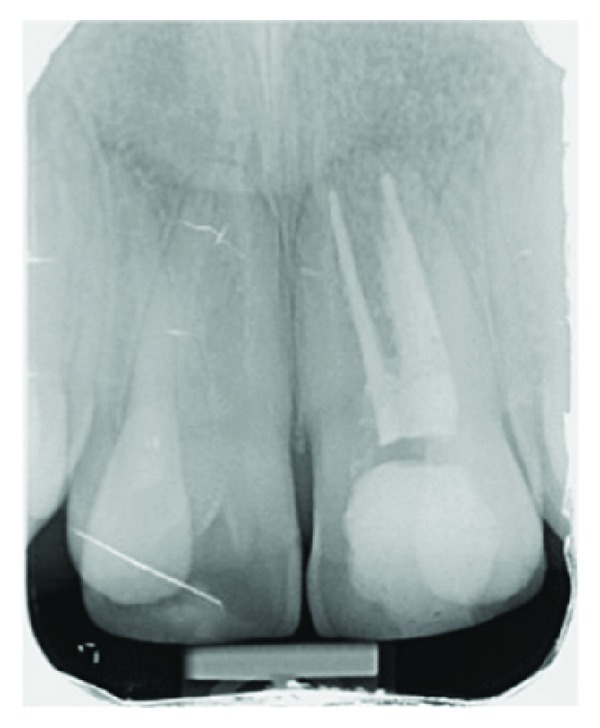
Postoperative final periapical radiograph of the left central incisor.

**Figure 7:**
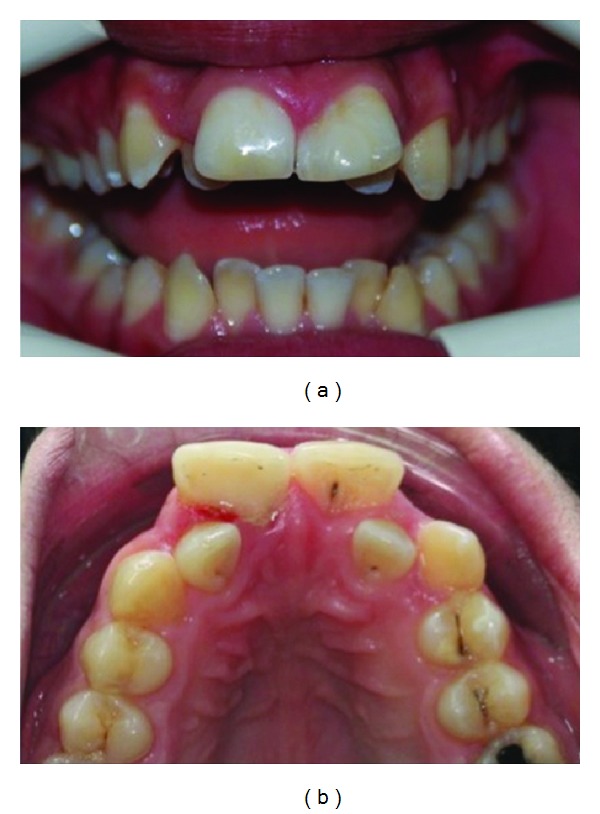
The appearance of teeth after restoration by nanofill composite resin material.

After administration of local anaesthesia to symptomatic tooth, rubber dam was applied for isolation. Caries lesion and the mainly affected talon cusp by the lesion were removed and an endodontic access cavity was prepared. Working length of the root canals was determined using ProPex II (Dentsply-Maillefer, Ballaigues, Switzerland). The root canals of geminated tooth were cleaned and shaped with ProTaper (Dentsply-Maillefer, Ballaigues, Switzerland) rotary instruments to size F5 to their full working lengths. During instrumentation root canals were irrigated with 2.5% sodium hypochlorite. After preparation, final irrigation was performed for one minute with 17% ethylenediaminetetraacetic acid and 2.5% sodium hypochlorite to remove the smear layer. The root canals were dried with sterile paper points and root canals were filled with using gutta-percha and AdSeal (Meta Biomed, Cheongju, Korea). And postoperative final radiograph was taken (Figure 6). After endodontic treatment tooth was restored with nanofill composite resin material using layering technique for aesthetic expectations (Figure 7).

The patient was recommended for clinical and radiographic followups. And he was referred to clinic of orthodontics due to the crowding and malocclusion.

## 3. Discussion

Fusion and gemination are morphological dental anomalies, which are difficult to distinguish. According to Levitas [7], counting teeth can make the differential diagnosis between fusion and gemination. If there is a missing tooth, anomaly can be termed as a fusion; if not it can be termed as a gemination. But it is not always possible in such cases like fusion with supernumerary tooth, or, if there is a congenitally absent tooth adjacent to the anomalous tooth, it can be misdiagnosed as a gemination [8]. Some authors submit observing the root morphology; others prefer to use the term of double teeth or use fusion and gemination as synonyms due to the uncertainty of the embryologic cause underlying the junction anomaly [9–11].

Geminated incisors generally have a single large pulp chamber and root canal, and division is usually incomplete as our patient's right incisor [12]. And left incisor of the patient has single large pulp chamber with mesial and distal two root canals. Tomazinho et al. [13] presented a geminated tooth with a single large pulp chamber and mesial and distal root canals. But differently from our case mesial and distal canals were joined at the apical third in his case. In the light of previous knowledge and clinical and radiographic findings, our case was diagnosed as bilateral gemination and left incisor diagnosed as gemination with talon cusp, which was affected by caries lesion.

The occurrence of talon cusp can cause clinical problems such as caries as in Tomazinho et al.'s [13] and our patient and also talon cusps can cause occlusal interference, displacement of affected tooth, irritation of tongue, and attrition [12].

Time of diagnosis changes the prognosis of teeth with talon cusp. In early diagnosis, only gradual grinding can be adequate. After grinding fluoride varnish has to be used and then concealed with resin composite to avoid postoperative sensitivity [14]. Our patient was diagnosed very late; therefore left central incisor had a deep carious lesion with pulp involvement and needed endodontic treatment.

Endodontic treatment of teeth that have rare malformations requires more attention during radiologic diagnosis and performing root canal treatment due to the unusual chamber and canal morphology [15]. Unusual morphology creates struggles during accessing pulp canal systems, determining working length and filling the large root canal. Especially in such cases taking aid from a three-dimensional CBCT scan has benefits before the endodontic treatment to estimate the root and canal morphology.

There are different treatment plans of geminated teeth in the literature. Sener et al. [6] performed minimal restorative and orthodontic treatment to improve the aesthetic appearance of the anterior teeth. Turkaslan et al. [16] implement prosthodontics restoration of all the six maxillary anterior teeth because of the wide and formless maxillary central incisors, while Gündüz and Açikgõz [12] decided to extract the geminated tooth before orthodontic treatment to provide space. As for us, we decided to perform nonsurgical endodontic treatment due to the deep carious lesion and then fixed appliance therapy to improve dental occlusion and aesthetic appearance.

The occurrence of gemination and talon cusp is very rare, but the clinicians should be conscious of the specific treatment needs, abnormal canal morphology, and importance of radiologic diagnosis. Different cases require alternative methods; therefore a multidisciplinary approach can supply success of the treatment plan.
